# Evaluation and Refinement of a Bank of SMS Text Messages to Promote Behavior Change Adherence Following a Diabetes Prevention Program: Survey Study

**DOI:** 10.2196/28163

**Published:** 2021-08-27

**Authors:** Megan MacPherson, Kaela Cranston, Cara Johnston, Sean Locke, Mary E Jung

**Affiliations:** 1 School of Health and Exercise Sciences Faculty of Health and Social Development University of British Columbia Kelowna, BC Canada; 2 Department of Kinesiology Brock University St Catharines, ON Canada

**Keywords:** text messaging, prediabetic state, telemedicine, telecommunications, exercise, diet, preventive medicine, mHealth, intervention development, behavior change, mobile phone

## Abstract

**Background:**

SMS text messaging is a low-cost and far-reaching modality that can be used to augment existing diabetes prevention programs and improve long-term diet and exercise behavior change adherence. To date, little research has been published regarding the process of SMS text message content development. Understanding how interventions are developed is necessary to evaluate their evidence base and to guide the implementation of effective and scalable mobile health interventions in public health initiatives and in future research.

**Objective:**

This study aims to describe the development and refinement of a bank of SMS text messages targeting diet and exercise behavior change to be implemented following a diabetes prevention program.

**Methods:**

A bank of 124 theory-based SMS text messages was developed using the Behaviour Change Wheel and linked to active intervention components (behavior change techniques [BCTs]). The Behaviour Change Wheel is a theory-based framework that provides structure to intervention development and can guide the use of evidence-based practices in behavior change interventions. Once the messages were written, 18 individuals who either participated in a diabetes prevention program or were a diabetes prevention coach evaluated the messages on their clarity, utility, and relevance via survey using a 5-point Likert scale. Messages were refined according to participant feedback and recoded to obtain an accurate representation of BCTs in the final bank.

**Results:**

76/124 (61.3%) messages were edited, 4/124 (3.2%) were added, and 8/124 (6.5%) were removed based on participant scores and feedback. Of the edited messages, 43/76 (57%) received minor word choice and grammar alterations while retaining their original BCT code; the remaining 43% (33/76, plus the 4 newly written messages) were recoded by a reviewer trained in BCT identification.

**Conclusions:**

This study outlines the process used to develop and refine a bank of SMS text messages to be implemented following a diabetes prevention program. This resulted in a bank of 120 theory-based, user-informed SMS text messages that were overall deemed clear, useful, and relevant by both individuals who will be receiving and delivering them. This formative development process can be used as a blueprint in future SMS text messaging development to ensure that message content is representative of the evidence base and is also grounded in theory and evaluated by key knowledge users.

## Introduction

### Background

The global prevalence of prediabetes is 375 million [1], which increases the likelihood of developing type 2 diabetes (T2D) by up to 70% [2]. Dietary and physical activity behavior change programs have been shown to prevent or delay the progression of prediabetes to T2D [3-5]. A recent meta-analysis that assessed the effects of mobile health (mHealth) diabetes prevention programs (including videos, web-based resources, videoconferencing, phone calls, and SMS text messaging) found that the use of technology-based diabetes prevention strategies led to clinically significant weight loss in individuals at risk of developing T2D [6].

With more than 5 billion mobile phone users worldwide [7] and more than 18 billion SMS text messages sent daily [8], SMS text messaging is one of the widest reaching mHealth interventions [7,9] and has been shown to improve dietary and physical activity behavior change [10-13]. Within diabetes care, text messaging has been shown to improve participants’ self-awareness, knowledge, and control over their condition and has been deemed a suitable technology for diabetes management; however, more research is needed to explore which message design features may be most effective in changing behaviors [14].

It is widely accepted that behavior change interventions should be developed using theory, past evidence, and formative research [15,16] and that they report sufficient detail regarding the intervention components [17,18]; however, many mHealth interventions lack rigor, theory, and the inclusion of experts in content development [6]. More specifically, few SMS text messaging interventions detail formative development and evaluation and typically lack information pertaining to content development [19-22], resulting in text messaging interventions with insufficient details to allow for replication or evaluation.

#### Prior SMS Text Message Development Research

Within the diabetes prevention literature, many SMS text messaging interventions do not provide information on how messages are developed. When formative development is described, messages are generally based on previous literature or health authority recommendations and are developed by a team of experts including clinicians, researchers, and individuals at risk of developing T2D [23-31]. Following message development by content experts, some studies take an additional step and have end users evaluate the messages to refine content based on key stakeholder preferences [23,25,26,29,32-34]. For example, O'Reilly and Laws [34] had participants rank messages as useful, unsure, or not acceptable and then conducted focus groups to improve messages rated as not acceptable. The use of theory within diabetes prevention messaging content is sparse, and only one study to date has linked their messages to behavior change techniques (BCTs) [23].

Although not in the field of diabetes prevention, Chai et al [35] used the Theoretical Domains Framework (TDF) and Behaviour Change Wheel (BCW) to integrate theory more effectively into SMS text messaging. The TDF was developed as a synthesis of 33 theories and 128 behavior change constructs combined into 14 distinct domains [36]. Following the identification of relevant domains within the TDF, Chai et al [35] used the BCW to identify relevant intervention functions and had a team of experts develop messages to map onto the three domains and four intervention functions they felt were relevant to their bank of messages. In phase 2 of their development, messages were reviewed by end users for message clarity, usefulness, and relevance. Once evaluated, the messages were refined based on participant responses before implementation and effectiveness testing.

#### This Work

**Figure 1:**
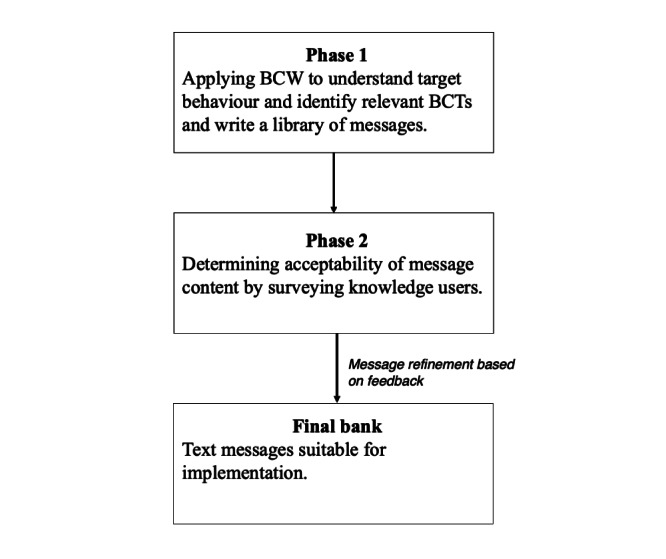
Text messaging content development and evaluation process. BCT: behavior change technique; BCW: Behaviour Change Wheel.

This study aims to outline the evaluation and refinement of a bank of SMS text messages designed for individuals at risk for T2D who have taken part in the Small Steps for Big Changes (SSBC) diabetes prevention program [37,38]. The iterative design used in the current text messaging content development included two broad phases: (1) application of the BCW framework to develop a bank of messages and (2) message evaluation and refinement by those representing SMS text message recipients and senders (Figure 1). Phase 1 has already been published (MacPherson et al [39]) but will be briefly described.

The BCW was developed using expert consensus and validation and has been used to design a myriad of behavior change interventions [40-44]. The BCW allows for the systematic and structured development of complex behavioral interventions and can improve subsequent implementation and evaluation through the selection of BCTs to target specific barriers and facilitators to engage in a behavior [45]. BCTs are the building blocks or *active components* within an intervention designed to modify a behavior. When developing the taxonomy of BCTs, Michie et al [45] identified 93 distinct BCTs, which can be grouped together in 16 different BCT categories.

During the message development phase, the research team identified the target behaviors of dietary and physical activity behavior change adherence. Barriers and facilitators to engage in these behaviors were identified through previous qualitative research conducted with individuals following their participation in the SSBC program [46]. Relevant BCTs were identified through systematic reviews addressing BCTs within diet or physical activity interventions for populations with or at risk of developing T2D [47-54] and BCTs currently being used within the SSBC diabetes prevention program [55]. All identified BCTs were assessed for inclusion by 2 members of the research team using the acceptability, practicability, effectiveness and cost-effectiveness, affordability, safety and side-effects, equity (APEASE) criteria [56]. Subsequently, messages were written pursuant to the included BCTs. This study identified 43 BCTs and 124 messages targeting diet and physical activity behavior change in individuals at risk of developing T2D. To improve intervention affordability and equity, messages were developed as one way messages, were limited to 160 characters (the maximum number of characters for a single SMS text message for most carriers—characters include but are not limited to numbers, letters, and spaces), and were at a less than an eighth-grade reading level.

## Methods

### Recruitment

A total of 25 potential knowledge users—5/25 (20%) diabetes prevention coaches and 20/25 (80%) past SSBC clients who consented to be contacted for future research studies—were contacted via email to participate in this study. Consenting individuals were asked to complete a web-based survey, which took approximately 60 minutes for diabetes prevention coaches (who were asked to review the entire message bank) and 20 minutes for past SSBC clients (who were asked to evaluate a random subset of approximately one-third of messages). Participants were compensated for their time with an electronic gift card in the amount of Can $40 (US $32.14) and Can $20 (US $16.07) for diabetes prevention coaches and SSBC clients, respectively.

### Procedures

Surveys were administered through a web-based survey platform (Qualtrics) in which study participants were asked to review messages on (1) readability or clarity, (2) usefulness, and (3) relevance by rating each message on a 5-point Likert scale (1=strongly disagree and 5=strongly agree). This process of message evaluation was used by Chai et al [35] and adapted from Redfern [57]. These questions were chosen as they target message acceptability, a key criterion within the APEASE criteria in assessing intervention content and delivery. This was built on the previous research stage, which assessed APEASE from the research team’s perspective, not end users’ perspective. For each message, following the quantitative questions, participants were provided with an open text box to provide additional feedback or suggestions regarding the message content.

At the conclusion of the survey, diabetes prevention coaches were asked how many months they would be willing to send messages (similar to the ones they reviewed) to their participants and how many per week they would be willing to send if they were required to do so without any automation. SSBC clients were similarly asked about the number of months they would like to receive messages and how many days per week they would find messages helpful.

Feedback regarding the prototype bank of messages was summarized, and messages were modified once Likert scale responses were addressed. A total sum score was created for each message by combining scores for all three questions (clarity, usefulness, and relevance), resulting in total sum scores per message ranging from 3 (if participants strongly disagreed for all questions) to 15 (participants strongly agreed for all questions). Before analysis, it was decided that any message with an average total sum score above 14 across all participants was to be retained with the option for revisions based on participant feedback. Total sum scores above 14 out of 15 would indicate that most end users rated the messages with a perfect score. Any message with a total score of ≤14 was to be refined to ensure that all messages to be used in the SSBC diabetes prevention program were highly rated by those sending and receiving the messages. This cutoff was chosen a priori to limit the impact that potential social desirability bias had on which messages were refined. Refinement of messages began with addressing any participant feedback; if a message with a score of ≤14 did not have any feedback, they were refined based on quantitative scores (eg, if a message had a lower readability score, it was altered to improve the clarity in which it was written). To ensure that all messages were accurately linked with BCTs, any revised messages were recoded by an independent reviewer for BCTs.

## Results

### Overview

A total of 18 adults—5/18 (28%) diabetes prevention coaches and 13/18 (72%) past SSBC clients—participated in the evaluation (7 females, 9 males, and 2 missing; mean age 47 years, SD 15), and an additional 7 past SSBC clients were contacted but did not participate in the survey. Participants were asked to provide demographic information including sex, age, ethnicity, highest level of education, occupation, and marital status (Table 1).

**Table 1:** Descriptive statistics for individuals that participated in the intervention.

Characteristics			All (N=18)		Diabetes prevention coaches (n=5)		Past Small Steps for Big Changes clients (n=13)
Age (years), mean (SD)			47.16 (15.21)		29.60 (4.72)		59.58 (6.32)
**Sex, n (%)**							
	Male	9 (50)		2 (40)		7 (54)	
	Female	7 (39)		3 (60)		4 (31)	
	Did not answer	2 (11)		0 (0)		2 (15)	
**Ethnic origin, n (%)**							
	White	13 (72)		3 (60)		10 (77)	
	Latin American	1 (6)		1 (20)		0 (0)	
	Asian	2 (11)		1 (20)		1 (8)	
	Indigenous	2 (11)		0 (0)		2 (15)	
**Annual income (Can $ [US $]), n (%)**							
	0-24,999 (0-19,987.58)	1 (6)		1 (20)		0 (0)	
	25,000-49,999 (19,988.38-39975.95)	1 (6)		1 (20)		0 (0)	
	50,000-74,999 (39,976.75-59,964.33)	4 (2)		1 (20)		3 (23)	
	75,000-99,999 (59,965.13-79,952.70)	4 (2)		1 (20)		3 (23)	
	≥100,000 (≥79,953.50)	7 (39)		1 (20)		6 (46)	
	Missing	1 (6)		0 (0)		1 (8)	
**Education, n (%)**							
	High school	2 (17)		0 (0)		2 (15)	
	College diploma	6 (33)		0 (0)		6 (46)	
	University degree	4 (22)		2 (40)		2 (15)	
	Postgraduate degree	6 (33)		3 (60)		3 (23)	
**Marital status, n (%)**							
	Single	4 (22)		3 (60)		1 (8)	
	Married	8 (44)		0 (0)		8 (62)	
	Common law	5 (28)		2 (40)		3 (23)	
	Divorced	1 (6)		0 (0)		1 (8)	

### Message Evaluation and Refinement

A total of 124 messages were included in the initial message bank. Each message was evaluated between 7 and 12 times, and messages received a total score of 13.77 (SD 0.76), with diabetes prevention coaches scoring messages higher than past SSBC clients (Table 2 provides scores on readability, relevance, and usefulness). On average, each message received approximately two comments (ranging from 0 to 5 comments per message).

**Table 2:** Mean scores for the message evaluations for diabetes prevention coaches, past Small Steps for Big Changes participants, and all participants combined.

Evaluation	All (N=18), mean (SD)	Diabetes prevention coaches (n=5), mean (SD)	Past Small Steps for Big Changes clients (n=13), mean (SD)
Total score (out of 15)	13.77 (0.76)	14.11 (0.97)	13.30 (0.92)
Readability (out of 5)	4.60 (0.30)	4.68 (0.39)	4.48 (0.30)
Relevance (out of 5)	4.60 (0.25)	4.73 (0.33)	4.40 (0.34)
Usefulness (out of 5)	4.58 (0.27)	4.70 (0.33)	4.41 (0.36)

A total of 65 messages received a score of ≥14 (Multimedia Appendix 1 [39,45] provides more information on individual message scores and BCTs). These highly rated messages primarily fall within the following BCT categories: *goals and planning* (16/65, 25%), *self-belief* (11/65, 17%), *repetition and substitution* (10/65, 15%), and *feedback and monitoring* (7/65, 11%). The remaining 59 messages were scored <14 and fell within the BCT categories: *self-belief* (10/59, 17%), *reward and threat* (9/59, 15%), *goals and planning* (8/59, 13%), and *identity* (7/59, 12%).

A total of 76 messages were edited (76/124, 61% of the initial message bank): 59 received a total score of ≤14 and were refined based on participant feedback and individual quantitative scores; 20 messages received a total score of >14 but had suggestions for improvement and were refined based on participant feedback. An additional 4 messages were added to the library based on participant suggestions, and 8 messages were removed as they received consistent comments regarding their applicability to the program by both diabetes prevention coaches and past SSBC clients. Of the 76 messages edited, 43/76 (57%) were altered based on minor word choice and grammatical errors and therefore retained their original BCT codes; the remaining 43% (33/76; plus the additional 4 messages that were written) were coded by a reviewer trained in BCT identification (Multimedia Appendix 2 [39,45] provides final message bank and corresponding BCTs).

The edited bank of messages (Multimedia Appendix 2) includes 120 messages based on 41 distinct BCTs. The BCT categories most used included *goals and planning* (26/120, 21.7%), *self-belief* (22/120, 18.3%), *repetition and substitution* (16/120, 13.3%), *social support* (11/120, 9.2%), and *natural consequences* (11/120, 9.2%). Table 3 provides information on all BCT categories within both the initial and edited message banks.

**Table 3:** Number of messages (%) within the initial and edited message bank within each of behavior change technique category.^a^

	Initial message bank scored ≥14 (n=65), n (%)	Initial message bank scored <14 (n=59), n (%)	Total initial message bank (n=124), n (%)	Edited message bank (n=120), n (%)
Goals and planning	16 (24.6)	8 (13.6)	24 (19.3)	26 (21.7)
Feedback and monitoring	7 (10.8)	1 (1.7)	8 (6.4)	6 (5)
Social support	4 (6.2)	6 (10.2)	10 (8.1)	11 (9.1)
Shaping knowledge	4 (6.2)	1 (1.7)	5 (4)	5 (4.1)
Natural consequences	6 (9.2)	3 (5.1)	9 (7.3)	11 (9.2)
Comparison of behavior	5 (7.7)	2 (3.4)	7 (5.7)	8 (6.7)
Associations	2 (3.1)	1 (1.7)	3 (2.4)	3 (2.5)
Repetition and substitution	10 (15.4)	6 (10.2)	16 (13)	15 (13)
Comparison of outcomes	4 (6.2)	5 (8.5)	9 (7.3)	10 (8.3)
Reward and threat	3 (4.6)	9 (15.3)	12 (9.7)	6 (5)
Regulation	2 (3.1)	0 (0)	2 (1.6)	3 (2.5)
Antecedents	4 (6.2)	3 (5.1)	7 (5.7)	5 (4.2)
Identity	1 (1.5)	7 (11.9)	8 (6.5)	8 (6.7)
Scheduled consequences	2 (3.1)	6 (10.2)	8 (6.5)	5 (4.2)
Self-belief	11 (16.9)	10 (16.9)	21 (16.9)	22 (18.3)
Covert learning	0 (0)	1 (1.7)	1 (0.8)	1 (0.8)

^a^Note that some messages had multiple behavior change techniques and thus may fall within more than one behavior change technique category.

Diabetes prevention coaches noted that if they were required to send similar messages to their own participants, they would be willing to send them for an average of 5 months (mode 1, median 3, range 1-12) and for an average of 3 messages per week (mode 3, median 3, range 1-5). When SSBC clients were asked about their preference for receiving messages, they indicated, on average, that they would prefer to receive messages for 7 months following program completion (mode 12, median 6, range 2-12) with 3 messages per week (mode 2, median 2, range 1-5).

## Discussion

### Principal Findings

The primary objective of this study was to outline the development, evaluation, and refinement process used to develop a bank of SMS text messages targeting health behaviors among individuals at risk of developing T2D. Message content development and refinement involved a range of diabetes prevention coaches, researchers, and past SSBC clients who reviewed content and provided suggestions to improve message content and clarity. Overall, diabetes prevention coaches and individuals who participated in the SSBC diabetes prevention program identified that the bank of messages was clear, relevant, and useful for individuals at risk of developing T2D. A total of 52.4% (65/124) of messages received a total sum score of ≥14 of 15. Of these 65 highly rated messages, 20/65 (31%) were physical activity messages, 19/65 (29%) were dietary messages, and 26/65 (40%) were general messages. Of those 59 messages scoring <14 of 15, 21/59 (36%) were physical activity messages, 22/59 (37%) were dietary, and 16/59 (27%) were general. Overall, the entirety of the message bank was rated 13.77 of 15, indicating general acceptability of most messages; however, the highest proportion of message scores of ≥14 contained general content, not targeting only diet or physical activity. Future research should examine whether general messages (eg, “Your first plan will not work 100% of the time. Continue to change your goals until you find what works best for you!”) versus targeted behavioral messages (eg, “Think about where, when and how you'll get your exercise in today!”) are more effective in behavior change adherence following the SSBC program.

### BCT Composition of the Message Bank

On the basis of total sum scores and participant feedback, 4 messages were added, 8 messages were removed, and 76 messages were edited based on knowledge user feedback, resulting in 120 theory-based, user-informed SMS text messages to be used following the SSBC diabetes prevention program. All 8 messages that were removed consisted of BCTs within the category *reward and threat*, for example, the lowest scoring message reads: “Did you put effort into meeting your exercise goals this week? Good for you! Don’t forget to reward yourself.” Messages similar to this one received consistent comments that this language and the use of external rewards were inconsistent with the program. The counseling portion of SSBC is informed by motivational interviewing, a collaborative communication style that draws on an individual’s own motivations and commitment to change. To stay consistent with the aims of SSBC to facilitate an individual’s autonomous motivation, messages including a focus on external motivators and rewards were removed. The final bank of messages includes either highly rated messages or messages that were tailored based on participant suggestions and relate to all 16 BCT categories. More than 70.8% (85/120) of this final message bank falls within five BCT categories: *goals and planning*, *self-belief*, *repetition and substitution*, *social support*, and *natural consequences*. Future research using this bank of messages can manipulate the BCT categories that are being used to provide additional experimental evidence for the categories that may be more or less effective in behavior change for individuals at risk of developing T2D.

Although BCTs within the categories of *goals and planning, feedback, and monitoring* have been cited as the most commonly used within physical activity behavior change interventions [58], there is not a large evidence base for which specific BCT categories or combinations of BCT categories may optimally influence long-term behavior change adherence because of lack of experimental evidence for different BCT categories. For example, Howlett et al [58] recently conducted a systematic review assessing BCTs within physical activity randomized controlled trials and found that studies including *biofeedback, demonstration of behavior, graded tasks, action planning, instruction on how to perform the behavior, prompts/cues, behavior practice,* and *self-reward* showed larger effect sizes compared with studies that did not. However, no BCTs within categories 11-16 (*regulation, antecedents, identity, scheduled consequences, self-belief*, or *overt learning*) were included in these analyses. The authors identified a total of 240 BCTs within the 26 included studies, with <3% corresponding to BCTs within the categories 11-16, thereby limiting researchers’ knowledge of whether or how these techniques may influence physical activity behavior change. Furthermore, researchers have shown that BCTs that are most frequently used in digital interventions may not adequately address user needs.

Asbjørnsen et al [59,60] provided a comprehensive analysis of both BCTs being consistently used, and the BCTs identified as relevant and meaningful to stakeholders to promote weight loss maintenance within digital interventions. The authors started by conducting a review that identified *feedback and monitoring, goals and planning, social support, shaping knowledge, associations,* and *repetition and substitution* as consistently applied BCT categories within digital interventions targeting weight loss motivation, adherence, and maintenance [60]. Following this, Asbjørnsen et al [59] conducted individual interviews and focus groups with end users to identify values and needs, as they relate to digital interventions targeting weight loss maintenance. On the basis of key values identified by end users, BCT categories identified as potentially relevant for digital interventions to support long-term behavior change maintenance included *goals and planning, feedback and monitoring, social support, self-belief, natural consequences,* and *identity.* This work highlights that although there is some overlap, BCTs that researchers are using do not necessarily overlap with what end users want or need to help them maintain long-term behavior change.

Although the primary BCT categories within the current message bank do not correspond to those that are commonly used in physical activity interventions [58], the systematic development and inclusion of key stakeholders may have resulted in more tailored BCT categories emerging as the most used. The inclusion of key stakeholders has been highlighted as an imperative step in designing digital interventions, as it allows the resulting intervention to reflect the values and support the goals identified by the end users [61,62].

### Limitations and Future Research

This paper outlines the iterative development of the content of SMS text messages; however, there is still an insufficient amount of research examining when or how frequently these messages should be sent and which specific messages or techniques are more effective than others. Although the inclusion of BCT mapping and knowledge users in the development and refinement of the bank of messages is a strength of this study, it is not yet known whether this bank of messages is effective in improving adherence to diet and physical activity recommendations following the SSBC diabetes prevention program. Future research should be conducted to identify further adaptations and refinements of the current bank of messages to facilitate long-term implementation. Such work could provide an in-depth understanding of *how* participants engage with the messages over time, which BCTs and specific content are most or least helpful, and how SMS text messages can influence behavior change over time.

Another limitation of this work is that content was tailored for deployment in SMS text messages, but study recruitment and assessment of the message bank was conducted via email and a web-based survey. Furthermore, as smartphones and associated apps for communication (eg, WhatsApp and Facebook Messenger) are becoming increasingly popular and traditional text messaging may become less accepted over time, possibly limiting the reach and usability of the current research program. That being said, the risk of T2D increases with age [63], and older adults are increasingly using text messaging [64] but are still less likely to own a smartphone compared with their younger counterparts [65], making text messaging a viable option for a diabetes prevention program. Furthermore, the current bank of messages can be easily adapted in future iterations for delivery across a myriad of platforms to improve accessibility and reach for varying populations.

It is important to note that on average, past SSBC clients rated messages lower than diabetes prevention coaches (Table 2). The precise rationale behind these ratings is not known. This could be because the responses of past SSBC clients are based on the knowledge of lived experience with the program; they may have a more accurate idea of how future SSBC clients will interact with the message bank and which content is most or least helpful from a participant standpoint. In addition, because of the training that diabetes prevention coaches receive and their overall passion for the subject matter, it is notable that differences in knowledge may have contributed to the overall augmented ratings of the bank of messages. Future studies could benefit from the contributions of past SSBC clients during the text messaging content development process. Despite these considerations, the message bank was rated favorably by both past SSBC clients and diabetes prevention coaches and as a result is believed to be suitable for implementation in the SSBC program.

Finally, given the large spread in the number of months and days per week, coaches were willing to send messages and participants wanted to receive them (ranging from 1 to 12 months of messages for 1 to 5 days per week), more empirical evidence is needed to identify the duration and frequency of messages that optimally influence behaviors following the SSBC program. In addition, although our group intends to implement and test this message bank following completion of the SSBC program, the final message bank may also be suitable for prospective or current participants, and future research can assess the time point at which these messages are most effective.

### Comparison With Previous Work

Many mobile phone text messaging interventions provide little to no detail regarding the processes used to develop their message content [19]. In many interventions, it is unclear whether content was developed ad hoc, if it was informed by behavior change theoretical frameworks, if content was reviewed by health experts, or whether feedback from stakeholders was sought to inform content. Although robust development of mobile behavior change interventions can be time consuming [66,67], this step is necessary to ensure that sufficient attention is placed on theoretical underpinnings and active intervention components, resulting in an mHealth intervention with a strong evidence base.

The process outlined in this paper includes the initial development of a bank of messages based on the BCW to link each message to a theoretical mechanism of action, followed by evaluation and refinement based on the opinions of researchers as well as those of past SSBC clients and diabetes prevention coaches. Broadly, the BCW has been used in the development of text messaging interventions [68], and the evaluation and refinement process has been successfully used in previous studies targeting parents and individuals with cardiovascular disease [35,57,69]. More specifically, the content of diabetes prevention SMS text messages typically includes the provision of information relating to diet and physical activity, reinforcement of key concepts following lessons, and reminders about goals and self-monitoring behavior; however, the authors cautioned the use of such mHealth interventions, as the marketplace is filled with products whose development lacks rigor, theory, and the inclusion of experts in content development [6].

Furthermore, to improve the quality and utility of mHealth interventions, it has been recommended that researchers specify not only theoretical mechanisms and provide explicit details on active intervention components [17,70] but that they also include knowledge users throughout the development process [16,71-74]. In this research program, message content was based on target barriers and facilitators identified by individuals who participated in the SSBC program [46], and content was further refined based on the preferences of those who represented those who would be receiving and sending the messages. Such collaborative approaches have been shown to improve the uptake of research into practice [75,76] and have been described as an integral part of program development to improve intervention relevance, impact, and efficiency [77].

### Conclusions

Understanding how to optimally intervene via SMS text messages requires rigorous development and consistent stakeholder engagement, which is often underreported [19-22]. This study reports on the iterative development and refinement of a bank of SMS text messages that are suitable for use or further effectiveness testing following the SSBC diabetes prevention program. Furthermore, the resulting bank of SMS text messages from this research has been written in a nonprogram-specific manner and can be implemented or tailored to other programs wishing to implement a text messaging intervention to improve adherence to behavior change. The authors request that any researchers planning to use this bank of messages acknowledge this paper in addition to the development paper [39]. The two-phase method used in this study to develop useful, clear, and relevant SMS text messages will provide a practical framework that can be used not only for future diabetes prevention research but also for other behavior change interventions. Overall, this study resulted in a bank of messages deemed acceptable by those who delivered and potentially received them. These messages are based on evidence and behavior change theory and have been refined based on feedback from those with lived experiences as diabetes prevention coaches and individuals who have participated in a diabetes prevention program.

## Appendix

Multimedia Appendix 1 Average and total sum scores per message.

Multimedia Appendix 2 Final message bank and associated behavior change technique.

## Abbreviations

APEASE: acceptability, practicability, effectiveness and cost-effectiveness, affordability, safety and side-effects, equity
BCT: behavior change technique
BCW: Behaviour Change Wheel
mHealth: mobile health
SSBC: Small Steps for Big Changes
T2D: type 2 diabetes
TDF: Theoretical Domains Framework
