# Supplementary material for: Evaluation and Refinement of a Bank of SMS Text Messages to Promote Behavior Change Adherence Following a Diabetes Prevention Program: Survey Study
Source: JMIR Form Res. 2021 Aug 27;5(8):e28163. doi: 10.2196/28163 (PMC8433931; doi:10.2196/28163)
Supplement: Multimedia Appendix 1 [file formative_v5i8e28163_app1.docx]

Appendix 1: Average and total sum score per message. Note that the total sum score is a sum of all three questions averaged across participants.

We request that any researchers planning to use this bank of messages reference/acknowledge this paper in addition to the development paper [MacPherson, M. M., Cranston, K. D., Locke, S. R., Bourne, J. E., & Jung, M. E. (2021). Using the behavior change wheel to develop text messages to promote diet and physical activity adherence following a diabetes prevention program. Translational Behavioral Medicine].

*Note that BCT numbers are those numbers assigned to each BCT within the BCT taxonomy v1 (Michie et al., 2013)

| **Message** | **Average score (/5)** | **Sum score (/15)** | **Behaviour Change Techniques (Behaviour change technique number*)** |
| --- | --- | --- | --- |
| Think about what goal you can set to get some exercise today. | 4.21 | 12.63 | Generalisation of target behaviour (8.6) |
| Make a goal to get some physical activity this week. | 4.24 | 12.71 | Social comparison (6.2) |
| Some people like to make small goals; think about what a good exercise plan for you would be this week. | 4.71 | 14.14 | Information about health consequences (5.1); Credible source (9.1) |
| Think about some ways you can fit exercise in when you have a busy week. | 4.76 | 14.29 | Information about health consequences (5.1) |
| Exercising while on vacation is hard! Think about ways you can be physically active while on vacation. | 4.70 | 14.10 | Instruction on how to perform the behaviour (4.1); Information about health consequences (5.1) |
| Each season has different challenges to staying active. Think about ways you can exercise next season when the weather is against you. | 4.52 | 13.57 | Focus on past success (15.3) |
| Think about where, when and how you'll get your exercise in today! | 4.83 | 14.50 | Graded tasks (8.7) |
| Remember to record your exercise in healthwatch360. | 4.62 | 13.86 | Social comparison (6.2); Habit formation (8.3) |
| Use the talk test to monitor exercise intensity. High intensity = you can't say 3 words. Moderate intensity = you can talk but can't sing. | 4.67 | 14.00 | Social support (unspecified; 3.1); Verbal persuasion about capability (15.1) |
| Sometimes we need the support of others to help us stick to our goals. Think about who in your life can help you stick to your exercise plan. | 4.60 | 13.79 | Review outcome goals (1.7) |
| Exercising with a buddy can give you motivation and support - and can be a nice distraction! | 4.76 | 14.29 | Self-monitoring of behaviour (2.3) |
| Scheduling your exercise sessions like an appointment can help you stay on track and get your workouts in! | 4.67 | 14.00 | Action planning (1.4) |
| Keep exercising! You might start to feel more energized & sleep better, and daily tasks might feel easier (like walking up or down stairs). | 4.60 | 13.80 | Social support (unspecified; 3.1); Instruction on how to perform the behaviour (4.1); Credible source (9.1) |
| Exercise lowers blood sugar levels right away - and for the next 24 hours! You can get these benefits with just a 15-minute walk. | 4.90 | 14.70 | Focus on past success (15.3) |
| If you haven’t exercised in a while, do something good for yourself by going for a 15-minute walk. It will affect your blood sugar levels right away! | 4.33 | 13.00 | Goal setting (behaviour; 1.1) |
| Pay attention to how you're feeling during exercise so that you know how hard you're working. | 4.71 | 14.14 | Goal setting (outcome; 1.3) |
| Many people find it challenging to stick to a new exercise routine. Think about some good reasons for you to stay with yours. | 4.57 | 13.71 | Problem solving (1.2) |
| Try going for a walk after dinner instead of sitting down to watch TV or scroll the internet. Just a 15-minute walk can make a difference! | 4.81 | 14.43 | Graded tasks (8.7) |
| Make exercise a part of your routine by linking it to something you do daily. Example, when I finish dinner then I will go for a walk. | 4.57 | 13.70 | Graded tasks (8.7) |
| Reversing old habits can be hard, so start small: try taking the stairs instead of the elevator, park at the far end of a parking lot, or commute by walking! | 4.71 | 14.14 | Problem solving (1.2) |
| Remember the exercise you did at the YMCA with your coach? Try to reach those same target heart rate zones on your own! | 5.00 | 15.00 | Action planning (1.4) |
| Start with shorter exercise sessions and work your way up over time! | 4.80 | 14.40 | Distraction (12.4) |
| Get at least 150 minutes of exercise per week; Canadian guidelines encourage us to reach this goal in sessions of 10 minutes or more. You can do it! | 4.86 | 14.57 | Action planning (1.4) |
| Continuing to exercise won’t always be easy. Remind yourself why you joined the program (we bet these reasons will outweigh reasons not to exercise!). | 4.74 | 14.22 | Prompts/cues (7.1) |
| You've been working hard! Think of how you can reward yourself if you meet your exercise goals this week. | 4.10 | 12.29 | Verbal persuasion about capability (15.1) |
| Did you put effort into meeting your exercise goals this week? Good for you! Don’t forget to reward yourself. | 3.81 | 11.43 | Behavioural substitution (8.2); Habit reversal (8.4) |
| For some people, regular exercise can decrease stress and improve their mood. | 4.70 | 14.10 | Problem solving (1.2); Action planning (1.4) |
| Need a distraction to get through your exercise session? Try exercising while listening to music or with a buddy. | 4.57 | 13.71 | Graded tasks (8.7) |
| Some people find that scheduling in their exercise can help reduce stress. | 4.67 | 14.00 | verbal persuasion about capability (15.1) |
| Try putting your running shoes and workout clothes on the kitchen counter. This can serve as a reminder to get your exercise in! | 4.43 | 13.29 | Behaviour substitution (8.2); Habit reversal (8.4) |
| Some people like to use distractions while exercising. Try listening to music or podcasts, or even exercising with a friend! | 4.83 | 14.50 | Review outcome goals (1.7) |
| Your behaviour is an example to those around you. When people see the effort you're putting into exercise, you're likely to inspire others! | 4.52 | 13.57 | Framing/reframing (13.2) |
| Thinking about how you're going to get 150 minutes of exercise in can be daunting. Instead, try thinking about reducing the time you spend sitting each day. | 4.48 | 13.43 | Self-monitoring of behaviour (2.3); Behavioural experiments (4.4) |
| It's not an all-or-nothing approach. Each day and week, you can get closer to your physical activity goals. Reward yourself for the little steps along the way! | 4.14 | 12.43 | Problem solving (1.2); Reducing negative emotions (11.2) |
| Each day, reward another step towards your goals. Today, reward yourself for exercising. Tomorrow, reward yourself for reaching your target heart rate! | 4.03 | 12.10 | Reduce negative emotions (11.2); Conserving mental resources (11.3) |
| You are capable of being active! You've already shown yourself how, so don't doubt your abilities. | 4.19 | 12.57 | Problem solving (1.2) |
| You've shown yourself that you can integrate exercise into your daily routine. Keep up the great work! | 4.71 | 14.14 | Social support (practical; 3.2); Distraction (12.4) |
| You got your heart rate into the zone and successfully exercised for 3 weeks as part of the program. Wow. Keep doing that on your own! | 4.17 | 12.50 | Pros and cons (9.2) |
| You've shown yourself that you are capable of being active! | 4.86 | 14.57 | Self-monitoring of behaviour (2.3) |
| Self-talk techniques may help you cope with physical sensations while exercising. Example: When I start to sweat, I will remind myself that this is normal. | 4.62 | 13.86 | Social comparison (6.2) |
| Think about all the positive benefits you will get during your next workout! | 4.38 | 13.14 | Social support (unspecified; 3.1); Focus on past success (15.3) |
| Think about what changes you can make so that half of your plate is filled with leafy greens. | 4.86 | 14.57 | Self-talk (15.4) |
| Think about ways you can cut out some added sugar from your diet. | 4.86 | 14.57 | Goal setting (outcome; 1.3) |
| Think about what small changes you can make to your diet this week. | 4.67 | 14.00 | Monitoring of emotional consequences (5.4) |
| Think about some ways you can eat healthy when you have a busy week. | 4.86 | 14.57 | Behavioural substitution (8.2); Habit reversal (8.4) |
| Eating healthy can be challenging at times (like at the holidays!). How can you increase how many vegetables you eat during these times? | 4.81 | 14.43 | Social support (unspecified; 3.1); Focus on past success (15.3) |
| Eating healthy can be challenging at times (like at the holidays!). Plan ahead and think of ways to reduce sugar during these times. | 4.53 | 13.58 | Verbal persuasion about capability (15.1) |
| Think about what you will do when you're craving refined carbs or sugar. | 4.57 | 13.70 | Self-monitoring of behaviour (2.3); Credible source (9.1) |
| Remember to record your food in healthwatch360. | 4.70 | 14.10 | Restructuring the physical environment (12.1); |
| Track your diet. This can help you understand your food choices and how to make changes in ways that are good for you. | 4.77 | 14.30 | Action planning (1.4) |
| Keep tracking your food. It can help you uncover the hidden sugar in your diet. | 4.80 | 14.40 | Graded tasks (8.7) |
| Having people who support us is important! Think about who in your life can help you stick to your food goals. | 4.60 | 13.80 | Non-specific reward (10.3); Self-reward (10.9); Reward approximation (14.4) |
| Continue tracking your food! Tracking your food can help you stay mindful of what you are putting in your body. | 4.87 | 14.60 | Information about social and environmental consequences (5.3); Social comparison (6.2) |
| Eat more vegetables and less sugar. This can decrease your risk of developing type 2 diabetes! | 4.90 | 14.70 | Focus on past success (15.3) |
| Many people like to log their food choices because it increases their accountability. | 4.96 | 14.89 | Information about emotional consequences (5.6) |
| Some people like to log their food while they are preparing it. Others like to log everything they eat at the end of the day. | 4.23 | 12.70 | Self-monitoring of behaviour (2.3) |
| Try swapping sugary foods for something else (like fruit instead of candy). | 4.60 | 13.80 | Behavioural experiments (4.4); Social comparison (6.2) |
| Try swapping refined carbs with above-ground veggies, which are better carb choices than below-ground veggies. | 4.03 | 12.10 | Focus on past success (15.3) |
| Some people find it helpful to check food labels for sugar content when they go grocery shopping. | 4.87 | 14.60 | Self-monitoring of behaviour (2.3) |
| Getting rid of old habits is hard, so start small: try eating fruit when you're craving something sweet, or drink water instead of juice/pop. | 4.80 | 14.40 | Action planning (1.4); Prompts/cues (7.1) |
| You and your coach probably talked about some of your eating habits in your sessions. You can still think about this on your own or with someone else. | 4.37 | 13.10 | Reduce negative emotions (11.2); Conserving mental resources (11.3) |
| Removing all sugar is hard! Start by reducing the amount or frequency of sugar. Example: Share your fave dessert with a friend or eat it less often. | 4.83 | 14.50 | Goal setting (outcome; 1.3) |
| Start with small changes to your carb choices and build on those successes! | 4.70 | 14.10 | Self-monitoring of behaviour (2.3) |
| The American Heart Association advises that women eat less than 6 tsp of added sugar and men eat less than 9 tsp of added sugar each day. | 4.53 | 13.60 | Self-incentive (10.7) |
| According to the Canadian Food Guide, you should fill half your plate with leafy greens! | 4.20 | 12.60 | Non-specific reward (10.3); Self-reward (10.9); Reward completion (14.5) |
| When eating healthy is feeling like a chore, think about the pros and cons to eating healthy. We'd guess that the pros outweigh the cons! | 4.33 | 13.00 | Verbal persuasion about capability (15.1) |
| Eating healthy won’t always be a priority. Think about why making these changes is important to you and how these reasons outweigh the cons. | 4.34 | 13.03 | Non-specific reward (10.3); Self-reward (10.9); Reward approximation (14.4) |
| Think about how you can reward yourself for eating healthy this week. The reward could be as simple as a pat on the back! | 4.11 | 12.33 | Self-monitoring of behaviour (2.3) |
| If you put in the effort to eat healthy this week, then think how about you can treat yourself in some way! | 4.30 | 12.90 | Self-talk (15.4) |
| Prepare food on the weekend or when you have extra time. This can help reduce the stress of making healthy meals when you're busy. | 4.77 | 14.30 | Information about health consequences (5.1) |
| Is it tempting to have unhealthy snacks in the house? If so, try putting them in a hard to reach spot so you will be less likely to go for them. | 4.48 | 13.44 | Social support (unspecified; 3.1) |
| Some people use food as a distraction and snack when they aren’t hungry. Think about some things you can do to distract or entertain yourself without snacks! | 4.53 | 13.60 | Behaviour substitution (8.2) |
| People in your life will see the healthy food choices you're making, and they may be inspired to do the same! | 4.40 | 13.20 | Social support (unspecified; 3.1) |
| It's very hard to think about taking away treats you enjoy. Instead, focus on getting in all your servings of fruits and veggies. | 4.53 | 13.60 | Non-specific reward (10.3); Self-reward (10.9); Reward approximation (14.4) |
| Don't forget to reward yourself for the small steps! Cutting out a little sugar each day can go a long way. | 4.70 | 14.10 | Self-talk (15.4) |
| Each day, reward another step towards your goals. Today, reward yourself for eating healthy. Tomorrow, reward yourself for cooking that healthy meal! | 3.88 | 11.64 | Social comparitson (6.2) |
| You can still be a healthy eater even if there have been days when you have indulged and gone off track. | 4.57 | 13.70 | Restructuring the physical environment (12.1); Restructuring the social environment (12.2); Distraction (12.4) |
| You've already proven to yourself that you can take steps towards a healthier diet. Keep up the good work! | 4.87 | 14.60 | Pros and cons (9.2) |
| You've already shown yourself that you are very capable of eating well and making healthier choices! | 4.90 | 14.70 | Behavioural practice/rehearsal (8.1); Habit formation (8.3) |
| Reducing the amount of sugar you eat isn't easy! Think about what has worked for you in the past to reduce your sugar intake. | 4.69 | 14.07 | Problem solving (1.2) |
| Before making a meal, remind yourself why you want to reduce sugar and refined carbs. | 4.40 | 13.20 | Verbal persuasion about capability (15.1) |
| There are many reasons why you should fill half your plate with leafy green veggies. Remind yourself of these reasons at your next meal! | 4.30 | 12.90 | Action planning (1.4) |
| Your first plan will not work 100% of the time. Continue to change your goals until you find what works best for you! | 4.87 | 14.60 | Credible source (9.1) |
| Your first plan will not work 100% of the time. Continue to change your goals until you find what works best for you! | 4.80 | 14.40 | Distraction (12.4) |
| Some people prefer to make small changes to either diet or exercise. Think about a plan that would work for you this week. | 4.87 | 14.60 | Framing/reframing (13.2) |
| Relapses are to be expected when making changes that you want to stick for a lifetime! Think about how you can respond to a relapse in a productive way. | 4.83 | 14.50 | Problem solving (1.2); Action planning (1.4) |
| When you are craving something sweet, focus on quality and quantity. Example: Only eat the best cookie in town - and only eat it once a week. :) | 4.44 | 13.33 | Problem solving (1.2); Action planning (1.4) |
| When you make a plan think about when, where, and how often you want to do a certain behaviour. | 4.57 | 13.70 | Identification of self as role model (13.1) |
| Make a plan for what you want to do this week for your health. The more detailed the plan, the more likely you are to do it! | 4.83 | 14.50 | Verbal persuasion about capability (15.1); Focus on past success (15.3) |
| Tracking your food/exercise can help you learn about your own behaviours. | 4.74 | 14.22 | Restructuring the physical environment (12.1); Avoidance/reducing exposure to cues for the behaviour (12.3) |
| While it may take a lot of time, tracking your food and exercise can make you feel more accountable - and can motivate you to continue making changes! | 4.67 | 14.00 | Framing/reframing (13.2) |
| Maintaining healthy behaviour can be challenging. Many people find it helpful to share this journey with a close friend. | 4.27 | 12.80 | Problem solving (1.2); Action planning (1.4) |
| Eat more leafy greens and less sugar, and exercise more! These simple actions have been shown to decrease the risk of type 2 diabetes. | 4.43 | 13.30 | Social support (unspecified; 3.1); Behvaioural substitution (8.2); Habit reversal (8.4) |
| Some people cut back on eating too much sugar, others exercise more. Take a second to think about how these goals can fit with what is important to you. | 4.74 | 14.22 | Identification of self as role model (13.1) |
| It can take a lot of trial and error to find what works. Many people feel that knowing what doesn't work for them is just as valuable as knowing what does! | 4.70 | 14.10 | Instruction on how to perform the behaviour (4.1); Information about health consequences (5.1) |
| People who make realistic changes that they enjoy are more likely to maintain them in the long run. | 4.70 | 14.10 | Prompts/cues (7.1) |
| Think about changes you can make for your health. Try substituting a behaviour you want to change with one that aligns with your goals. | 4.15 | 12.44 | Identification of self as role model (13.1) |
| Reminders can be super helpful when we are trying to form new habits. Put a reminder in your phone, a note on your fridge, or get creative! | 4.81 | 14.44 | Self-talk (15.4) |
| Kicking old habits is not easy, but we know you can do it! Think about new habits you can form to slowly replace old ones. | 4.44 | 13.33 | Review outcome goals (1.7); Non-specific reward (10.3); Self-reward (10.9) |
| We call ourselves Small Steps for Big Changes for a reason. Remember that those small steps can amount to something great! | 4.89 | 14.67 | Imaginary reward (16.2) |
| Remember: making small changes that build over time is more effective than one big change you can’t stick with. | 4.85 | 14.56 | Generalisation of target behaviour (8.6) |
| Track your diet and exercise. Research shows this is an important part of changing behaviour for many people. | 4.70 | 14.11 | Pros and cons (9.2) |
| Research shows that making changes to your diet and exercise is more effective than drugs in preventing type 2 diabetes! | 4.93 | 14.80 | Information about health consequences (5.1); Salience of consequences (5.2) |
| Think about the pros of all the changes you are making - they probably greatly outweigh the cons! | 4.57 | 13.71 | Pros and cons (9.2) |
| Don't forget to reward yourself for the progress you’ve made in achieving your goals! | 4.59 | 13.78 | Self-talk (15.4) |
| Reflect on the progress you've made. You should reward yourself for all of this hard work you're putting in to staying healthy! | 4.39 | 13.17 | Non-specific reward (10.3); Self-reward (10.9) |
| Make a goal for this week. If you make an effort to achieve that goal, be sure to reward yourself! | 4.67 | 14.00 | Social support (unspecified; 3.1) |
| Take a moment to think about all the hard work you've put in. You are becoming a healthier you! | 4.74 | 14.22 | Goal setting (outcome; 1.3) |
| Planning ahead can help reduce stress when you're busy. | 4.77 | 14.30 | Social comparison (6.2) |
| Set yourself up for success. Make small changes to your home or work that make it easier to make the choices that align with your goals. | 4.70 | 14.11 | Goal setting (outcome; 1.3) |
| You've worked hard and shown yourself what you are capable of. You can inspire others, too! What an amazing achievement. :) | 4.05 | 12.14 | Credible source (9.1) |
| You are a role model to those around you. The steps you take to staying healthy and active can improve the health of those around you! | 4.44 | 13.33 | Framing/reframing (13.2) |
| Not every part of changing behaviours is fun. Try to focus on the positive aspects of making these changes! | 4.20 | 12.60 | Verbal persuasion about capability (15.1); Focus on past success (15.3) |
| Don’t think of a slip as harmful or bad; they are INEVITABLE. Don't be discouraged! What is more important is how we react to slips and recommit to change. | 4.78 | 14.33 | Social support (unspecified; 3.1); Focus on past success (15.3) |
| Making lifestyle changes takes time. Even if you aren't yet at your goal, celebrate all the progress you've made! | 4.63 | 13.89 | Behaviour substitution (8.2) |
| Take some time to think about and celebrate each new change you've made. | 4.67 | 14.00 | Non-specific reward (10.3); Self-reward (10.9); Reward approximation (14.4) |
| Diet and exercise changes are exciting and challenging. You are taking steps to be healthier and can continue to succeed in these changes! | 4.63 | 13.90 | Non-specific incentive (10.6); Self-incentive (10.7) |
| When you put your mind to something, you do it! | 4.70 | 14.11 | Non-specific incentive (10.6); Self-incentive (10.7) |
| You are capable of making lasting changes to improve your health! | 4.81 | 14.44 | Social support (unspecified; 3.1); Identification of self as role model (13.1) |
| You've shown yourself that you can make these changes on your own! | 4.52 | 13.56 | Behaviour substitution (8.2) |
| Think about all the great changes that you have made since you joined the program. Amazing! | 4.70 | 14.10 | Non-specific reward (10.3); Self-reward (10.9); Reward approximation (14.4) |
| You've done this before, you can do it again! | 4.03 | 12.10 | Focus on past success (15.3) |
| You are making changes that will have benefits for your overall health. Remind yourself of these benefits. | 4.59 | 13.78 | Non-specific reward (10.3); Self-reward (10.9); Reward approximation (14.4) |
| Remind yourself why having a healthy lifestyle is important. Keep these reasons in mind as you go about your day! | 4.72 | 14.16 | Non-specific reward (10.3); Self-reward (10.9); Reward approximation (14.4) |
