# Supplementary material for: Evaluation and Refinement of a Bank of SMS Text Messages to Promote Behavior Change Adherence Following a Diabetes Prevention Program: Survey Study
Source: JMIR Form Res. 2021 Aug 27;5(8):e28163. doi: 10.2196/28163 (PMC8433931; doi:10.2196/28163)
Supplement: Multimedia Appendix 2 [file formative_v5i8e28163_app2.docx]

Appendix 2: Final message bank and associated Behaviour Change Technique (BCT).

We request that any researchers planning to use this bank of messages reference/acknowledge this paper in addition to the development paper [MacPherson, M. M., Cranston, K. D., Locke, S. R., Bourne, J. E., & Jung, M. E. (2021). Using the behavior change wheel to develop text messages to promote diet and physical activity adherence following a diabetes prevention program. Translational Behavioral Medicine].

*Note that BCT numbers are those numbers assigned to each BCT within the BCT taxonomy v1 (Michie et al., 2013)

| **Overall Message Focus** | **Message** | **Behaviour Change Techniques (Behaviour change technique number*)** |
| --- | --- | --- |
| Diet | Think about what changes you can make so that half of your plate is filled with leafy greens. | Goal setting (behaviour; 1.1) |
| Diet | Think about ways you can cut out some added sugar from your diet. | Goal setting (outcome; 1.3) |
| Diet | Think about small changes you can make to your diet this week. | Goal setting (outcome; 1.3) |
| Diet | Think about some ways you can eat healthy when you have a busy week. | Problem solving (1.2) |
| Diet | Eating healthy can be challenging at times (like during the holidays!). Think about ways to ensure half your plate is filled with veggies. | Problem solving (1.2); Action planning (1.4) |
| Diet | Eating healthy will be challenging at times. Plan ahead to reduce sugar intake (e.g., only order 1 dessert to share with a friend on vacation). | Problem solving (1.2); Action planning (1.4) |
| Diet | Eating healthy will be challenging at times so plan ahead. For example, during the holidays stick to your favourite cookie instead of trying one of everything. | Problem solving (1.2); Action planning (1.4) |
| Diet | Make a plan for the next time you're craving refined carbs or sugar (example: when I crave candy, then I will find a low sugar option to satisfy my craving). | Problem solving (1.2); Action planning (1.4) |
| Diet | Some people find it helpful to record their food. If this is you, try writing it down on a notepad or use an app (e.g, healthwatch360 or myfitness pal). | Self-monitoring of behaviour (2.3) |
| Diet | Tracking your diet can help you see the ingredients (like sugar) in your food choices and decide if/how you want to make changes to your diet. | Behavioural experiments (4.4) |
| Diet | Tracking your food can help you uncover the hidden sugar in your diet. | Behavioural experiments (4.4) |
| Diet | Continue tracking your food! Tracking your food can help you stay mindful of what you are putting in your body. | Self-monitoring of behaviour (2.3) |
| Diet | Eating more vegetables and less sugar can decrease your risk of developing type 2 diabetes! | Information about health consequences (5.1) |
| Diet | Many people like to log their food choices because it increases their accountability. | Social comparison (6.2) |
| Diet | Some people like to track their food before they make it, others like to log everything they eat at the end of the day. Find what works for you! | Social comparison (6.2) |
| Diet | Everyone has cravings! Think of a way to satisfy your craving in while eating less sugar (e.g., swap candy for fruit). | Behaviour substitution (8.2) |
| Diet | Try swapping refined carbs (e.g., white bread) with above-ground veggies (e.g., cucumber). | Behaviour substitution (8.2) |
| Diet | Some people find it helpful to check food labels for sugar content when they go grocery shopping. | Social comparison (6.2); Habit formation (8.3) |
| Diet | Getting rid of old habits is hard, so start small: try eating fruit when you're craving something sweet, or drink water instead of juice/pop. | Behaviour substitution (8.2); Habit reversal (8.4) |
| Diet | You and your coach probably talked about eating habits. Keep thinking about these healthy choices on your own or with someone else. | Generalisation of target behaviour (8.6) |
| Diet | Removing all sugar is hard! Start by reducing the amount or frequency of sugar. Example: Share your fave dessert with a friend or eat it less often. | Graded tasks (8.7) |
| Diet | Start with small changes to your carb choices and build on those successes! | Graded tasks (8.7) |
| Diet | To reduce diabetes risk, it is recommended that women eat less than 6 tsp of added sugar and men eat less than 9 tsp of added sugar each day. | Credible source (9.1) |
| Diet | According to the Canadian Food Guide, you should fill half your plate with above ground veggies (e.g., spinach, cucumber, and cauliflower to name a few)! | Credible source (9.1) |
| Diet | When eating healthy feels challenging, think about the pros and cons to eating healthy. We'd guess the pros outweigh the cons! | Pros and cons (9.2) |
| Diet | Eating healthy won’t always be a priority. Think about why making these changes is important to you and if these reasons outweigh the cons. | Pros and cons (9.2) |
| Diet | If you put in the effort to eat healthy this week, then think about how you can celebrate this success! | Non-specific reward (10.3); Self-reward (10.9) |
| Diet | Try preparing food on the weekend or when you have extra time. This can help reduce the stress of making healthy meals when you're busy. | Problem solving (1.2); Reducing negative emotions (11.2) |
| Diet | Are sugary snacks in the house too tempting? Some people get rid of snacks in the house, others put them somewhere hard to reach (out of sight out of mind). | Restructuring the physical environment (12.1); Avoidance/reducing exposure to cues for the behaviour (12.3); Social Comparison (6.2) |
| Diet | We all use food as a distraction at times and snack when we are bored. Think about something you can do to entertain yourself without snacks! | Distraction (12.4) |
| Diet | The healthy food choices we make can inspire others around us to do the same! | Identification of self as role model (13.1) |
| Diet | It can be hard to think about taking away treats you enjoy. Instead, focus on getting in all your servings of fruits and veggies. | Framing/reframing (13.2) |
| Diet | Don't forget to celebrate the small steps you make! Cutting out a little sugar each day can go a long way. | Non-specific reward (10.3); Self-reward (10.9); Reward approximation (14.4) |
| Diet | You are still a healthy eater even when there are days that don't go according to plan. Make your next meal a balanced and healthy one! | Verbal persuasion about capability (15.1) |
| Diet | You've already proven to yourself that you can take steps towards a healthier diet. | Verbal persuasion about capability (15.1); Focus on past success (15.3) |
| Diet | You've already shown yourself that you are very capable of eating well and making healthier choices! | Focus on past success (15.3) |
| Diet | Reducing the amount of sugar you eat isn't easy! Think about what has worked for you in the past to reduce your sugar intake. | Focus on past success (15.3) |
| Diet | When you are grocery shopping or before making a meal, remind yourself why you want to reduce sugar and refined carbs. | Self-talk (15.4) |
| Diet | Don't deny your cravings! When you are craving something sweet, focus on quality and quantity. Example: when you want a cookie make it the best cookie in town! | Problem solving (1.2); Action planning (1.4) |
| General | Your behaviour is an example to those around you. When people see the effort you are putting into creating a healthy lifestyle, you're likely to inspire others! | Identification of self as role model (13.1) |
| General | It's the small steps that lead to big changes in your health. Every step you take this week is a step towards a healthier you. | Social support (unspecified) (3.1); Verbal persuasion about capabilities (15.1) |
| General | Having people who support us is important! Think about who in your life can help you stick with the goals that are important to you. | Social support (unspecified; 3.1) |
| General | Your first plan will not work 100% of the time. Continue to change your goals until you find what works best for you! | Review outcome goals (1.7) |
| General | Think about what small steps you can take to reach your health goals. | Goal setting (behaviour; 1.1) |
| General | Some people find it easier to make small changes to their diet or exercise. Think about a plan to make a small change that would work for you this week. | Action planning (1.4) |
| General | There will be times when you don't achieve your goals. This happens to everyone! Think about how you can respond to these times in a productive way. | Problem solving (1.2) |
| General | When you make a plan think realistically about when, where and how often you want to do a certain behaviour. | Action planning (1.4) |
| General | Make a plan for what you want to do this week for your health. The more detailed the plan, the more likely you are to do it! | Action planning (1.4) |
| General | Tracking your food/exercise can help you learn about your own behaviours. | Self-monitoring of behaviour (2.3) |
| General | Tracking your food and exercise can take a lot of time, but it can make you feel more accountable and motivated to continue making changes! | Problem solving (1.2) |
| General | Maintaining diet and exercise behaviours can be challenging. Many people find it helpful to share this journey with a close friend. | Social support (unspecified; 3.1) |
| General | Eat more leafy greens and less sugar, and exercise more! These actions have been shown to decrease the risk of type 2 diabetes. | Instruction on how to perform the behaviour (4.1); Information about health consequences (5.1) |
| General | Some people cut back on eating too much sugar, others exercise more. Take a second to think about how these goals can fit with what is important to you. | Social comparison (6.2) |
| General | It can take a lot of trial and error to find what works. Many people feel that knowing what doesn't work for them is just as valuable as knowing what does! | Behavioural experiments (4.4); Social comparison (6.2) |
| General | People who make realistic changes that they enjoy are more likely to maintain them in the long run. | Information about social and environmental consequences (5.3); Social comparison (6.2) |
| General | Making changes to diet and exercise wont always be fun or easy. Think about reasons to stick with it. | Information about social and environmental consequences (5.3); Info about emotional consequences (5.6); Pros and Cons (9.2) |
| General | Reminders can be helpful when we are trying to form new habits. Put a reminder in your phone, a note on your fridge, or get creative! | Prompts/cues (7.1) |
| General | We call ourselves Small Steps for Big Changes for a reason. Remember that those small steps can amount to something great! | Graded tasks (8.7) |
| General | Remember: making small changes that build over time is more effective than one big change you can’t stick with. | Graded tasks (8.7) |
| General | Track your diet and exercise. Research shows this is an important part of changing behaviour for many people. | Self-monitoring of behaviour (2.3); Credible source (9.1) |
| General | Research shows that making changes to your diet and exercise is more effective than prescription drugs in preventing type 2 diabetes! | Information about health consequences (5.1); Credible source (9.1) |
| General | Think about all the great reasons why you are making these changes - these reasons probably outweigh the cons! | Pros and cons (9.2) |
| General | Change is challenging. Take time to reflect on your dedication - be proud of yourself! | Focus on past success (15.3) |
| General | Don't forget to celebrate the progress you've made in achieving your goals! | Non-specific reward (10.3); Self-reward (10.9); Reward approximation (14.4) |
| General | Reflect on the progress you've made. You will surprise yourself on how far you've come! | Focus on past success (15.3) |
| General | Take a moment to think about all the hard work you've put in. You are becoming a healthier you! | Social support (unspecified; 3.1); Focus on past success (15.3) |
| General | Planning ahead can help reduce stress when you're busy. | Reduce negative emotions (11.2); Conserving mental resources (11.3) |
| General | Set yourself up for success. Make small changes to your home or work to help you succeed in achieving your goals. | Restructuring the physical environment (12.1); |
| General | You've worked hard and proved to yourself what you are capable of. Take pride in your daily achievements. You can inspire others, too! | Social support (unspecified; 3.1); Identification of self as role model (13.1) |
| General | The steps you take to staying healthy and active can improve the health of those around you! | Identification of self as role model (13.1) |
| General | Not every part of changing behaviours is fun. Try to focus on the positive aspects of making these changes (e.g., improved health, increased energy)! | Framing/reframing (13.2) |
| General | Don’t think of a slip as harmful or bad; they are INEVITABLE. Don't be discouraged! What is more important is how we react to slips and recommit to change. | Framing/reframing (13.2) |
| General | Celebrate all the progress you've made, the goals you've accomplished and the goals you will conquer! | Non-specific reward (10.3); Self-reward (10.9); Reward approximation (14.4) |
| General | Take some time to think about and reflect on how you feel about each new change you've made. | Focus on past success (15.3) |
| General | Time some time to think about and celebrate each new change you've made. | Non-specific reward (10.3); Self-reward (10.9); Reward completion (14.5) |
| General | Diet and exercise changes are exciting and challenging. You are taking steps to be healthier. Continue to succeed in these pride-worthy changes! | Verbal persuasion about capability (15.1) |
| General | When you put your mind to something, you do it! | Verbal persuasion about capability (15.1) |
| General | You are capable of making lasting changes to improve your health! | Verbal persuasion about capability (15.1) |
| General | Think about all the great changes that you have made since joining the program. Every small step you take along the way counts! | Focus on past success (15.3) |
| General | You are making changes that will benefit your overall health. Remind yourself of these benefits. | Self-talk (15.4) |
| General | Remind yourself why having a healthy lifestyle is important. Keep these reasons in mind as you go about your day! | Self-talk (15.4) |
| Physical Activity | Think about what exercise goal you want to make today, if any. | Goal setting (outcome; 1.3) |
| Physical Activity | Make a goal to do some physical activity this week. | Goal setting (outcome; 1.3) |
| Physical Activity | Some people like to make short term goals; think about what would be a good exercise plan for you this week. | Goal setting (outcome; 1.3) |
| Physical Activity | Think about some ways you can fit exercise in when you have a busy week. | Problem solving (1.2) |
| Physical Activity | Exercising while on vacation is hard! Think about ways you can be physically active while on vacation. | Action planning (1.4) |
| Physical Activity | Each season has different challenges to staying active. Think about ways you can exercise when the weather changes. | Problem solving (1.2); Action planning (1.4) |
| Physical Activity | Think about where, when and how you'll get your exercise in today! | Action planning (1.4) |
| Physical Activity | Consider recording your exercise in a phone app or notebook. | Self-monitoring of behaviour (2.3) |
| Physical Activity | Use the talk test to monitor exercise intensity. High intensity = it's difficult to talk. Moderate intensity = it's difficult to sing. | Self-monitoring of behaviour (2.3) |
| Physical Activity | We all need support from others to help us with our goals sometimes. Think about who in your life can help you stick to your exercise plan. | Social support (unspecified; 3.1) |
| Physical Activity | Exercising with a buddy can give you motivation and support - and can be a nice way to spend time together! | Social support (practical; 3.2) |
| Physical Activity | Scheduling your exercise sessions like an appointment can help you stay on track and get your workouts in! | Action planning (1.4); Prompts/cues (7.1) |
| Physical Activity | Keep exercising! Regular exercise has many benefits, including increased energy levels and better sleeps. | Information about health consequences (5.1) |
| Physical Activity | Exercise lowers blood sugar levels right away - and for the next 24 hours! You can get these benefits with just a 15-minute walk. | Information about health consequences (5.1) |
| Physical Activity | You can do something good for your body by going for a 15-minute walk. It will have a positive impact on your blood sugar levels right away! | Information about health consequences (5.1); Salience of consequences (5.2) |
| Physical Activity | Pay attention to how you're feeling during exercise so that you know how hard you're working. | Monitoring of emotional consequences (5.4) |
| Physical Activity | Many people find it challenging to stick to a new exercise routine. Think about some good reasons to stick to yours. | Social comparison (6.2) |
| Physical Activity | Consider reducing your sitting time by going for a walk after dinner - just a 15-minute walk can make a difference! | Behavioural substitution (8.2); Habit reversal (8.4) |
| Physical Activity | Make exercise a part of your routine by linking it to something you do daily. For example, when I finish dinner I will go for a walk. | Behavioural practice/rehearsal (8.1); Habit formation (8.3) |
| Physical Activity | Reversing old habits can be hard, so start small: try taking the stairs instead of the elevator, park at the far end of a parking lot, or commute by walking! | Behavioural substitution (8.2); Habit reversal (8.4) |
| Physical Activity | Remember the exercise you did with your coach? Try to reach those same target heart rate zones on your own! | Generalisation of target behaviour (8.6) |
| Physical Activity | If you have missed a few exercise sessions, it's okay! Start with a shorter exercise session and work your way up over time! | Graded tasks (8.7); potentially Social support unspecified (3.1) |
| Physical Activity | Canadian guidelines encourage us to get at least 150 minutes of exercise per week. You can do it! | Social support (unspecified; 3.1); Instruction on how to perform the behaviour (4.1); Credible source (9.1) |
| Physical Activity | Continuing to exercise won’t always be easy. Remind yourself why you joined the program (we bet these reasons will outweigh reasons not to exercise!). | Pros and cons (9.2) |
| Physical Activity | For some people, regular exercise can decrease stress and improve their mood. | Information about emotional consequences (5.6) |
| Physical Activity | Need a distraction to get through your exercise session? Some people like to listen to music or exercise with a buddy! What works for you? | Restructuring the physical environment (12.1); Restructuring the social environment (12.2); Distraction (12.4) |
| Physical Activity | Some people find that scheduling in their exercise can help reduce stress. | Reduce negative emotions (11.2); Conserving mental resources (11.3) |
| Physical Activity | Try putting your workout clothes and shoes near the front door. This can serve as a reminder to get your exercise in! | Prompts/cues (7.1) |
| Physical Activity | Some people like to use distractions while exercising. If that’s you, try listening to music or podcasts, or ever exercising with a friend! | Distraction (12.4) |
| Physical Activity | There are some weeks when getting 150 minutes of exercise seems daunting. In those weeks, try thinking about reducing the time you spend sitting each day. | Framing/reframing (13.2) |
| Physical Activity | It's not an all-or-nothing approach. If you partially complete your goal next week it will bring you closer to your long-term physical activity goals. | Social support (unspecified) (3.1); Verbal persuasion about capabilities (15.1) |
| Physical Activity | Every exercise session you complete gets you closer to your long term goals. Remember to celebrate the small steps you're making along the way! | Non-specific reward (10.3); Reward approximation (14.4) |
| Physical Activity | You have the ability to live an active lifestyle, you've already shown yourself how! Remember how capable you are if you start to doubt your abilities. | Verbal persuasion about capability (15.1); Focus on past success (15.3) |
| Physical Activity | You've shown yourself that you can integrate exercise into your daily routine. Keep up the great work! | Social support (unspecified; 3.1); Focus on past success (15.3) |
| Physical Activity | You got your heart rate into the zone and successfully exercised for 3 weeks as part of the program. You are capable of being active on your own! | Verbal persuasion about capabilities (15.1); Focus on past success (15.3) |
| Physical Activity | You've shown yourself that you are capable of being active! | Focus on past success (15.3) |
| Physical Activity | We all feel different physical sensations while exercising. When you start to sweat or breath heavy, remind yourself that it is a normal part of exercise. | Self-talk (15.4) |
| Physical Activity | Think about all the positive benefits you will get during your next workout (better mood, sleep, reducing your diabetes risk)! | Imaginary reward (16.2); Information about health consequences (5.1) |
| Physical Activity | Try substituting a behaviour you want to change with one that aligns with your goals (eg, I want to stay healthy for my family so I take the stairs instead of the elevator). | Behaviour substitution (8.2) |
